# Enteric Pathogens in HIV/AIDS from a Tertiary Care Hospital

**DOI:** 10.4103/0970-0218.55291

**Published:** 2009-07

**Authors:** Beena Uppal, Bineeta Kashyap, Preena Bhalla

**Affiliations:** Department of Microbiology, Maulana Azad Medical College, New Delhi, India

**Keywords:** Bacteriology, diarrhea, diagnosis, HIV, parasitology

## Abstract

**Background:**

Patterns of enteric infections in HIV in developing countries may differ in several important ways from developed countries, the knowledge of which can often guide therapy when resource limitations hamper the exact diagnosis of the etiological agent in HIV-associated diarrhea.

**Objectives:**

The primary objective of this study was to define and compare the microbial etiologies of diarrhea in HIV-1 infected and non infected patients and in HIV infected non diarrheal patients.

**Materials and Methods:**

This study was conducted between April 2007 and July 2007 at the Department of Microbiology, Maulana Azad Medical College, New Delhi. Stool samples from 50 HIV seropositive cases with diarrhea (study group), 50 HIV seropositive cases without diarrhea (control group I), and 50 HIV seronegative cases with diarrhea (control group II) were examined. After the diagnosis of HIV infection was made, routine parasitological and bacteriological detection was done. An ELISA was used for the detection of Clostridium difficile toxin and Cryptosporidium antigen in stool samples.

**Results:**

The overall prevalence of enteric parasitosis in the study group was 20% and the bacteria identified were Escherischia coli in 24% of the case, Clostridium difficile in 10% of the cases, Salmonella species and Vibrio cholerae in 4% of the cases, and Shigella species in 2% of the cases. Candida species was identified in 36% of the cases.

**Conclusions:**

Identification of the etiological agent of diarrhea in a patient with AIDS is very important as it can help in the institution of appropriate therapy and the reduction of morbidity and mortality in these patients.

## Introduction

HIV/AIDS (Human Immunodeficiency Virus/Acquired Immunodeficiency Syndrome) is a major problem in India with more than 6 million recorded cases by the end of 2005.(1) Patients with HIV are prone to developing a panorama of diseases during their lifetime. AIDS represents the most severe squeal of immunosuppression caused by HIV and is a constellation of diseases reflecting the late manifestation of HIV infection. Immunosuppression caused by HIV infection leads to the development of severe opportunistic infections and otherwise rare tumors. Among them, diarrhea is a significant cause of morbidity observed in a majority of studies.(2,3) In fact, it is the second leading cause of hospital visits in developing nations and makes its place in the top ten worldwide.(4) Reports indicate that diarrhea occurs in 30-60% of patients with AIDS in developed countries and in about 90% of such patients in developing countries.(5) In tropical countries, chronic diarrhea associated with weight loss (slim disease) is often the presenting illness of HIV-1 infected individuals.(6) This diarrhea wasting syndrome in association with a positive HIV-1 serology test is an AIDS-defining criterion, according to the World Health Organization (WHO) classification.(7)

HIV infection and AIDS are fast becoming a major threat in India. Patterns of enteric infections in developing countries like ours, where hygiene is poor and intercurrent infection rates are high, may differ in several important ways from patterns of developed countries. Knowledge of the pattern of the infection can often guide therapy when resource limitations hamper the exact diagnosis of the etiological agent in HIV-associated diarrhea. The primary objectives of this study were to define the microbial etiologies of diarrhea in HIV-1 infected individuals and compare them with those of HIV seronegative diarrheal patients and HIV-1 infected individuals without diarrhea.

## Materials and Methods

### Study population and study design

This study was conducted prospectively between April 2007 and July 2007 at the HIV and Diarrhea and Parasitology Laboratory, Department of Microbiology, Maulana Azad Medical College attached to Lok Nayak Hospital, New Delhi, which is a tertiary care hospital with 1500 beds catering to the health needs of people in Delhi and the adjoining states of North India.

After receipt of informed consent from patients and approval from the ethical committee, 150 stool samples were taken as follows:

Fifty stool samples from HIV seropositive cases with diarrhea (study group).

Fifty stool samples from HIV seropositive cases without diarrhea (control group I).

Fifty stool samples from HIV seronegative cases with diarrhea (control group II).

Each patient was interviewed using a questionnaire concerning general demographic characteristics, type of illnesses, and clinical symptoms.

### Inclusion criteria

An episode of diarrhea was defined as the occurrence of an average of at least three liquid bowel movements daily for at least 1 week before the visit to the hospital.

### Exclusion criteria

Persons who had received antibiotics/antiparasitic treatment for diarrhea within the past 14 days were excluded.

### Laboratory studies

Diagnosis of HIV infection and microbiological diagnosis of enteric infection in stool samples were done in all 150 cases.

### Diagnosis of HIV infection

The diagnosis of HIV infection was done by following the standard protocol at our voluntary counseling and testing center (VCTC) that employs pre test and post test counseling and obtains informed consent before HIV testing. Three different immunoassays (Micro ELISA, J. Mitra and Co. Pvt. Ltd, India; Retroquic, Qualpro Diagnostics, India; and Capillus, Trinity Biotech, USA) were used following the standard manufacturer's instructions.

### Diagnosis of enteric infections

Stool specimens were collected fresh and processed in the Diarrhea and Parasitology Laboratory within 4 hours of collection. Parasite detection was done by direct examination after formalin-ether-concentration of stool specimens as wet saline and iodine mount techniques for the detection of protozoan trophozoites and cysts and helminthic eggs and larva. Additionally, all samples were subjected to Gram stain and a modified acid-fast stain for coccidian oocysts.(8)

A part of the stool sample was processed for the detection of bacterial pathogens using selective and differential media as per standard protocol.(9)

For detection of *Clostridium difficile* toxin in stool samples, the *Clostridium difficile* Toxin A + B Antigen Detection Microwell ELISA (IVD Research Inc., Carlsbad, CA 92008 USA) was used following the manufacturer's instructions.

For detection of the Cryptosporidium antigen in stool samples, the Cryptosporidium Antigen Detection Microwell ELISA (IVD Research Inc., Carlsbad, CA 92010 USA) was used following the manufacturer's instructions.

## Results

This prospective study was conducted between April 2007 and July 2007. A total of 150 patients were examined in this study; 50 from HIV seropositive cases with diarrhea (study group), 50 from HIV seropositive cases without diarrhea (control group I), and 50 from HIV seronegative cases with diarrhea (control group II).

The age and gender profile of the study and the control subjects is presented in Table 1. In the study group, the age range of 31-40 years old was the most predominant in overall size (46%). The age range of 21-30 years old and 0-10 years old had the maximum number of cases with 42% and 78% in control groups I and II, respectively. Males outnumbered females in the study group and control group II subjects with a male to female ratio of approximately 1:4 and 3:2, respectively. However, no such gender difference was observed in control group I.

**Table 1:** Age and gender distribution of the study and control subjects

Age groups (years)	HIV seropositive with diarrhea study group (n=50)				HIV seropositive without diarrhea control group I (n=50)				HIV seronegative with diarrhea control group II (n=50)			
			
	Males	Females	Total	%	Males	Females	Total	%	Males	Females	Total	%
0-10	2	2	4	8	1	1	2	4	23	16	39	78
11-20	0	0	0	0	4	1	5	10	2	3	5	10
21-30	10	6	16	32	5	16	21	42	1	1	2	4
31-40	12	11	23	46	11	6	17	34	3	0	3	6
41-50	3	1	4	8	2	1	3	6	0	0	0	0
51 +	2	1	3	6	2	0	2	4	1	0	1	2
Total	29	21	50	100	25	25	50	100	30	20	50	100

From the study, the overall prevalence of enteric parasitosis in the study group, control group I, and control group II were found to be 20%, 2%, and 18%, respectively. The overall prevalence of enteric parasites in males was 6% (9/150) and 7.3% (11/150) in females. The proportion of protozoan parasites in the study group was 70% as compared with 100% and 66.7% in control groups I and II, respectively. The distribution of different enteric parasites in the study group and control subjects is depicted in Table 2.

**Table 2:** Prevalence of specific intestinal parasites among the study and control subjects

Parasites detected	HIV seropositive with diarrhea study group (n=50) frequency (%)	HIV seropositives without diarrhea control group I (n=50) frequency (%)	HIV seronegative with diarrhea control group II (n=50) frequency (%)
*Ascaris lumbricoides*	2 (20)	0	2 (22.2)
Hookworm	0	0	1 (11.1)
*Hymenolopsis nana*	0	0	0
*Entamoeba histotytica*	2(20)	0	1 (11.1)
*Giardia lamblia*	2(20)	0	5 (55.5)
*Blastocystis hominis*	0	0	0
*Cryptosporidium parvum*	2 (20)	1 (100)	0
*Isospora belli*	1 (10)	0	0
*Cyclospora cayetanensis*	0 (0)	0	0
*Strongyloides stercoralis*	1 (10)	0	0
*Trichuris trichuria*	0	0	0
Total	10	1	9

The bacteria identified in the stool of HIV seropositive individuals with diarrhea were *Escherischia coli* in 12 (24%) cases, *Clostridium difficile* in 5 (10%) cases, Salmonella species in 2 (4%) cases, *Vibrio cholerae* in 2 (4%) cases, and Shigella species in 1 (2%) case. In HIV seropositive individuals without diarrhea, *Escherischia coli* again was the most predominant bacteria identified in stool samples (10%) followed by *Clostridium difficile* in 4% cases. In HIV seronegative individuals with diarrhea, whereas *Clostridium difficile* was not seen, *Escherischia coli* was found in 22% of the cases. In addition, *Vibrio cholerae* was isolated in 26% of the cases with 69.2% and 30.8% belonging to Ogawa and Inaba subtypes, respectively [Table 3].

**Table 3:** Enteric microbial pathogens other than parasites identified in stool samples of study and control subjects

Pathogen other than parasite	HIVseropositive with diarrhea study group (n=50) number (%)	HIV seropositive without diarrhea control group I (n=50) number (%)	HIV seronegative with diarrhea control group II (n=50) number (%)
Candida species	18 (45)	6 (46.2)	1 (4)
Salmonella species	2 (5)	0	0
Shigella species	1 (2.5)	0	0
*Vibrio cholerae*			
Ogawa	2 (5)	0	9 (36)
Inaba	0	0	4(16)
*Clostridium difficile*	5 (12.5)	2 (15.4)	0
Enteropathogenic *Escherishia coli*	8 (20)	3 (23.1)	9 (36)
Enteroinvasive *Escherishia coli*	4 (10)	2 (15.4)	2 (8)
Total	40	13	25

As far as fungal pathogens are concerned, the Candida species was identified in 36%, 12%, and 2% of the cases in the study group, control group I, and control group II subjects, respectively.

## Discussion

Although HIV/AIDS and water-borne infections, exemplified by diarrhea, are the leading causes of morbidity and mortality in developing countries, their association has received only cursory attention. Identification of the etiological agent of diarrhea in a patient with AIDS is very important as it can help in the institution of appropriate therapy and the reduction of morbidity and mortality in these patients. This study was therefore conducted to ascertain the scope and frequency of potential enteric bacterial pathogens isolated from stool samples of HIV-positive and -negative individuals with and without diarrhea. The etiology for such diarrhea could be parasitic, bacterial, fungal, enteric viruses, or HIV itself may contribute to diarrhea. In addition to microbes, other factors such as medication, immune deregulation, autonomic dysfunction, and nutritional supplementation play a substantial role in diarrhea of patients with HIV/AIDS.(5) Shigellosis, Campylobacter infections, and Cryptosporidiosis occur relatively more frequently in HIV-1 infected persons than in persons without HIV-1 infection. Some agents produce diarrhea almost exclusively in HIV-1 infected persons (e.g., *Mycobacterium avium* complex, Cytomegalovirus, and HIV-1enteropathy). Others cause more severe, more prolonged, or more often recurrent diarrhea in the presence of HIV-1 infection (e.g., Cryptosporidiosis species, Isospora species, Salmonella species, Astrovirus, Adenovirus, Calcivirus and perhaps Microsporidium species, *Cyclospora cayetanensis*, Shigella species, and Campylobacter species). Some agents apparently have unaltered courses but occur commonly in HIV-1 infected persons (e.g., *Clostridium difficile*).(10) Among persons with HIV infection, 40-80% of diarrheal illnesses have an identifiable cause and a bacterial etiology is common.(11,12) Diarrheal illnesses due to parasites in patients infected with HIV is on the rise. Many studies have highlighted the emergence of important protozoan parasites and helminths as a major cause of morbidity and mortality in patients with AIDS.(2,13,14) In resource limited countries such as India, enteric infections remain common in the general population and for those people infected with HIV, with geographic differences in the reported prevalence of individual pathogens reflecting differences in pathogen prevalence, standards of hygiene, and diagnostic methods used.(2) There are many reports regarding the frequency of various pathogens causing diarrhea from different parts of India.(2,15) Some studies also demonstrated regional variability of pathogens,(16) as well as changing trends of etiology in the same population.(11) With the myriad of etiologies and sometimes-altered natural history of enteric infections in HIV-1 infected persons with diarrhea, it remains uncertain how well clinical manifestations or risk factors predict microbial etiology or are able to guide the empirical choice of therapy for an individual patient.

In our study 31-40 years old, 21-30 years old, and 0-10 years old were the most predominant age groups in overall size affected with 46%, 42%, and 78% of the cases, respectively. Previous studies have also indicated HIV prevalence to be most common in the 21-30 year old age group(17,18) and in the non HIV infected group, diarrhea is more commonly seen in children than in adults in developing countries. Our study shows a male preponderance in HIV infected patients with diarrhea similar to previous studies;(20) however, others have reported more females than males in this population.(17)

We found an overall prevalence of enteric parasitosis in the study group, control group I, and control group II to be 20%, 2%, and 18%, respectively. A previous study reported that a potential enteric pathogen was isolated from all (100%) of the HIV-positive individuals with diarrhea and 68 (52.3%) without diarrhea.(17) Another study reported a prevalence of enteric parasitosis of 36% and 14% in patients with HIV with and without diarrhea, respectively,(18) whereas others have reported a higher percentage of enteric parasitosis in Indian patients with HIV with diarrhea.(19) Studies in Africa have reported a prevalence of intestinal parasitosis varying from 19 to 33%.(21,22) A recent study conducted in Chennai reported 13% enteric parasitosis among HIV-positive individuals and normal individuals without diarrhea.(23) In our study, the overall prevalence of enteric parasites in males was 6% (9/150) and 7.3% (11/150) in females. A previous study from Nepal reported 56% and 44% enteric parasitosis in male and female groups of patients with HIV/AIDS, respectively.(24)

Our study shows that the proportion of protozoan parasites in the study group was 70% as compared with 100% and 66.7% in control groups I and II, respectively. Previous authors have also reported a higher prevalence of protozoa as compared with helminthes in enteric parasitosis(18,22,24) in HIV/AIDS. In HIV-positive patients with diarrhea, out of 10 cases of enteric parasitosis, we isolated helminthes in 3 cases (*Ascaris lumbricoids* in 2 and *Strongyloides stercoralis* in 1) and protozoa in 7 cases (*Entamoeba histolytica, Giardia lamblia*, and *Cryptosporidium parvum* in 2 each and Isospora belli in 1). In HIV-positive patients without diarrhea, we isolated only *Cryptosporidium parvum* in 1 case. In HIV-negative individuals with diarrhea, out of 9 cases of enteric parasitosis, helminthes were found in 3 cases (*Ascaris lumbricoids* in 2 and *Ancylostoma duodenale* in 1) and protozoa were detected in 6 cases (*Entamoeba histolytica* in 1 and *Giardia lamblia* in 5). These findings suggest that patients with HIV/AIDS had more intestinal parasite infections than the control groups and the only parasites clearly more prevalent in this population were *Cryptosporidium parvum, Isospora belli,* and *strongyloides stercoralis*. A recent study from North India reported that of the emerging parasites, *Isospora belli* was most frequently detected followed by *Cryptosporidium; Blastocystis hominis* and *E. histolytica* were the most frequent conventional pathogen followed by *Giardia lamblia.*(25) Few other Indian studies have also reported *Isospora belli* followed by *Cryptosporidium parvum* as the most common pathogen among enteric parasites in HIV associated diarrhea.(19,23) The high rate of infection with *Isospora belli* poses a threat to HIV-positive patients. Diagnosed cases of *Isospora belli* were considerably fewer in this study, which could possibly be either due to more sensitive detection methods used in studies reporting a higher prevalence or a reflection of low prevalence in this study population. The actual rate of this infection in immunocompetent individuals and patients with AIDS is likely to be underestimated due to asymptomatic shedding of oocysts and treatment with trimethoprim - sulphamethoxazole for other infections in AIDS cases, which may confer some protection against this parasite. Other studies have reported *Cryptosporidium parvum* as the most common pathogen among enteric parasites in the HIV/AIDS population.(12,24,26) HIV opportunistic infections, cryptosporidiosis inclusive, tend to vary from one locality to another and from one country to the another depending on the level of contamination in the water, food, and contact with animals, which are important factors in the dissemination of the parasite. Finally, the small unfilterable size (3-5 Lm) of oocysts, their resistance to chlorine disinfections, and low infective dose are the major infective potential of *Cryptosporidium parvum*. No *Microsporidia* and *Cyclospora* were detected in our study similar to a few other studies,(25) though other studies from India have reported a low prevalence of cyclosporidiasis(15,18,23) and microsporidiosis.(19,23) Thorough investigations on a large number of patients are required to know the exact role of these pathogens in HIV-related diarrhea in India. Our findings tend to support the view that the more ‘common’ parasites (*Ascaris lumbricoides, Ancylostoma duodenale, Giardia lamblia*, and *Entamoeba histolytica*) are not opportunistic in patients with AIDS and identification of these common parasites in up to 60% of patients with AIDS and the controls is a reflection of poor environmental hygiene. Until recently, more frequently-associated parasites with diarrhea were: *Giardia lamblia, Entamoeba histolytica, Balantidium coli,* etc. Since the onset of the AIDS epidemic, the number of parasitic pathogens recognized and the frequency with which they are encountered in clinical practice have increased. These parasites can cause self-limiting diarrhea of short duration in healthy individuals, but in the immunocompromised host, including patients with AIDS, diarrhea is usually chronic and, sometimes, life-threatening. The high proportion of patients with AIDS who had diarrhea in the absence of identified parasite infections strongly indicates the existence of other diarrheaogenic agents or mechanisms. The detection of these will require more comprehensive and better controlled studies.

On bacterial culture of stool, the common organisms associated with diarrhea in HIV-infected individuals in our study were *Escherichia coli* (30%), *Clostridium difficile* (12.5%), Salmonella species (5%), *Vibrio cholerae* (5%), and Shigella species (2.5%). Whereas only *Escherichia coli* (38.5%) and *Clostridium difficile* (15.4%) were found in the stool of HIV-infected individuals without diarrhea, the stool of HIV seronegative individuals with diarrhea showed *Vibrio cholerae* and *Escherichia coli* in 52% and 44% of the total bacterial isolates in this group. Previous studies in India have also reported the isolation of similar organisms with slightly variable frequencies in the stool of such patients;(18,23) however, in one of these studies, no bacterial pathogens other than *Escherichia coli* and Klebsiella species were isolated from stool specimens of HIV-positive individuals without diarrhea and normal individuals without diarrhea.(23) Another study from Thailand found that the isolation rates of bacterial enteropathogens causing diarrhea in AIDS patients with diarrhea (APD) (18%, 62/350) were considerably lower than those in non AIDS patients with diarrhea (NAPD) (43%, 152/350) (*P*<0.05).(27)

As far as fungal pathogens are concerned, we isolated the *Candida* species in 18 cases (36%), 6 cases (12%), and 1 case (2%) of the 50 cases each in the study group, control group I, and control group II, respectively. In a previous study in India, the *Candida* species was identified in 2.6% of the cases consisting of one case in acute diarrhea, one in control group and two cases in chronic diarrhea.

## Conclusion

Enteric parasitosis might be one of the major health problems among patients infected with HIV, particularly those with AIDS. Studies from various parts of the world show contrasting prevalence rates with marked geographical variations.(28,29) This emphasizes the need for thorough investigations of these patients to identify pathogens for proper management. Opportunistic enteric pathogens, for which there is no effective treatment, the emergence of new opportunistic infections, and the enlarging pattern of drug resistance continues to be a challenging task. However, better understanding of HIV-1-induced mucosal immunosuppression, sound clinical management, careful diagnostic evaluation, the development of newer antimicrobial agents, and judicious patient management should help to meet this challenge and may help to reduce morbidity and untimely mortality for patients with HIV/AIDS in India.

## References

[1] (2004). NACO report on AIDS in India.

[2] Joshi M, Chowdhary AS, Dalar PJ, Maniar JK (2002). Prevalence of intestinal parasitic pathogens in HIV-seropositive individuals in Northern India. Natl Med J India.

[3] Mwachari C, Batchelor BI, Paul J, Waiyaki PG, Gilks CF (1998). Chronic diarrhea among HIV-infected adult patients in Nairobi, Kenya. J Infect.

[4] Attili SV, Gulati AK, Singh VP, Varma DV, Rai M, Sundar S (2006). Diarrhea, CD4 counts and enteric infections in a hospital - based cohort of HIV-infected patients around Varanasi, India. BMC Infect Dis.

[5] Framm SR, Soave R (1997). Agents of diarrhoea. Med Clin North Am.

[6] Colebunders R, Lusakumuni K, Nelson AM, Gigase P, Lebughe I, van Marck E (1990). Persistent diarrhoea in Zairian AIDS patients: An endoscopic and histologic study. Gut.

[7] Dallabetta G, Miotti P (1992). Chronic diarrhoea in AIDS patients in the tropics: A review. Trop Doct.

[8] Arrowood M, Sterling C (1989). Comparison of conventional staining methods and monoclonal antibody-based methods for Cryptosporidium oocyst detection. J Clin Microbiol.

[9] Farmer II, Murray P, Baron EJ, Pfaller M, Tenover F, Yolken RH (1995). Enterobacteriaceae: Introduction and identification. Manual of clinical microbiology.

[10] Carcamo C, Hooton T, Wener MH, Weiss NS, Gilman R, Arevalo J (2005). Etiologies and manifestations of persistent diarrhea in adults with HIV-1 infection: a case-control study in Lima, Peru. J Infect Dis.

[11] Call SA, Heudebert G, Saag M, Wilcox CM (2000). The changing etiology of chronic diarrhea in HIV patients, with CD4 less than 200/mm^3^. Am J Gastroenterology.

[12] Chhin S, Harwell J, Bell JD, Rozycki G, Ellman T, Barnett JM (2006). Etiology of chronic diarrhea in antiretroviral-naive patients with HIV infection admitted to norodom sihanouk hospital, Phnom Penh, Cambodia. Clinical infectious diseases.

[13] Gagandeep K (2000). Opportunistic protozoan parasitic infections of the gastrointestinal tract. Indian J Med Microbiol.

[14] Nwokediuko SC, Ozumba UC (2002). Intestinal helminthes in relation to chronic diarrhoea in HIV-seropositive adults in Enugu. Niger Postgrad Med J.

[15] Mohandas, Sehgal R, Sud A, Malla N (2002). Prevelence of intestinal parasitic pathogens in HIV-seropositive individuals in Northern India. Jpn Jn Inf Dis.

[16] Lynen L, Biot M (2001). Clinical aids care guidelines for resource poor settings. Medecins sans frontieires.

[17] Obi CL, Ramalivhana J, Momba MN, Igumbor J (2007). Scope and frequency of enteric bacterial pathogens isolated from HIV/AIDS patients and their household drinking water in Limpopo Province. Water SA.

[18] Kumar SS, Ananthan S, Lakshmi P (2002). Intestinal parasitic infection in HIV infected patients with diarrhoea in Chennai. Indian J Med Microbiol.

[19] Dalvi S, Mehta P, Koticha A, Gita N (2006). Microsporidia as an emerging cause of parasitic diarrhoea in HIV seropositive individuals in Mumbai. Bombay Hosp J.

[20] Sapkota DA, Ghimire PA, Manandhar S (2004). Enteric Parasitosis in Patients with Human Immunodeficiency Virus (HIV) Infection and Acquired Immunodeficiency Syndrome (AIDS) in Nepal. Journal of Nepal Health Research Council.

[21] Chintu C, Luo C, Baboo S, Khumalo-Ngwenya B, Mathewson J, DuPont HL (1995). Intestinal Parasites in HIV-seropositive Zambian Children with Diarrhoea. J Trop Pediatr.

[22] Sarfati C, Bourgeois A, Menotti J, Liegeois F, Moyou-Somo R, Delaporte E (2006). Prevalence of intestinal parasites including Microsporidia in Human Immunodeficiency Virus-infected adults in Cameroon: a cross-sectional study. Am J Trop Med Hyg.

[23] Kumar SS, Ananthan S, Saravanan P (2002). Role of coccidian parasites in causation of diarrhea in HIV infected patients in Chennai. Indian J Med Res.

[24] Sapkota Da, Ghimire Pa, Manandhar S (2004). Enteric parasitosis in patients with Human Immunodeficiency Virus (HIV) infection and Acquired Immunodeficiency Syndrome (AIDS) in Nepal. Journal of Nepal Health Research Council.

[25] Prasad KN, Nag VL, Dhole TN, Ayyagari A (2000). Identification of Enteric Pathogens in HIV-positive Patients with Diarrhoea in Northern India. J Health Popul Nutr.

[26] Adesiji YO, Lawal RO, Taiwo SS, Fayemiwo SA, Adeyeba OA (2007). Cryptosporidiosis in HIV infected patients with diarrhea in Osun state southwestern Nigeria. Eur J Gen Med.

[27] Suthienkul O, Aiumlaor P, Siripanichgon K, Eampokalap B, Likhanonsakul S, Utrarachkij F (2001). Bacterial causes of Aids-associated diarrhea in Thailand. Southeast Asian J Trop Med Public Health.

[28] Wilcox CM (2000). Etiology and evaluation of diarrhea in AIDS: A global perspective at the millennium. World J Gastroenterol.

[29] Sanchez TH, Brooks JT, Sullivan PS, Juhasz M, Mintz E, Dworkin MS (2005). Bacterial Diarrhea in Persons with HIV Infection, United States, 1992-2002. Clin Infect Dis.

